# Abstract of the Proceedings of the Buffalo Medical Association

**Published:** 1863-01

**Authors:** J. F. Miner

**Affiliations:** Secretary


BUFFALO

### Medical and Surgical Journal

VOL. II. JANUARY, 1863. NO. 6.

### ORIGINAL COMMUNICATIONS.

ART. I.—*Abstract of the Proceedings of the Buffalo Medical Association.*

TUESDAY EVENING, December 2d, 1862.

Prof. James P. White, President, in the Chair.

*Dr. Gay* exhibited seven stones and the handles of two pocket knives taken from the intestines of a man who had died suddenly. Post mortem examination ordered by the Coroner; Drs. Eastman and Fanner present, with Dr. Gay. The larger stones were an inch and a half in their long, by an inch in their short diameter, and would weigh from one to two ounces each. It was said that the man had been in the habit of swallowing such articles as stones, knives, swords, &c. for several years, for the amusement and astonishment of spectators and for the purpose of obtaining a living, by contributions. It was supposed, by the stones being found somewhat on the way in passage, that they had been swallowed several days; one large stone was found in the transverse colon. They had produced death by inducing inflammation.

*Dr. White* remarked that the time, they had probably been in the intestinal tube, was interesting, as showing how many similar substances will sometimes pass the bowels with impunity. Recolects seeing a "bull's-eye watch," owned by a very truthful sea captain, who assured him that at one time, being captured in South America, and desiring to save his watch, he swallowed it. After several days it passed, and he washed it clean and swallowed it the second time. Mentioned this as suggestive of a useful practical lesson—better to pass foreign substances into the stomach, if lodged in the throat or œsophagus, than to make violent effort to extract them. Passage of various articles through the intestinal canal are frequently reported. Dr. Storck had recently reported a case to this Society where large numbers of pins and needles had passed with impunity.—Other physicians had at various times reported such cases to the Society. The proper treatment to adopt was to wait patiently for their passage; active cathartics were very injurious.

*Dr. Eastman* called attention to one important point of interest. The very limited extent of inflammation present in the case examined by Dr. Gay, and that, not of the adhesive character usually seen in such cases. They concluded before finding the stones, and handles, that they had caused inflammation in their passage, but had not been retained. Had never seen a case with so little inflammation where foreign bodies had lodged and produced it. Almost all substances capable of being swallowed may pass the intestinal tube. A few years since he used to give cathartics for removal of such articles as copper cents, buttons, &c., &c., but soon learned that it was wholly unnecessary. In the case reported that evening there was great thickening of the mucus coat of the stomach, and but very little inflammation.

*Dr. Rochester* remarked that Dr. Dayton had not long since sent him a four-bladed knife which had passed the intestinal canal of a patient of his; the sides of the handle were of pearl. and had separated from the lining; they passed also not long afterwards. Recently Dr. Dayton brought him a patient who had swallowed his teeth, two or three in number, clasped on either side of the mouth; they were pushed down and soon passed from the bowels; it passed readily, without much trouble.

*Dr. Rochester* would, as a matter of record, remind the Association that when recently reporting a case of pyocele he had spoken of a case of hæmatocele, and promised to report the results. It came into his hands from his predecessor in service in the Sisters' Hospital, Dr. Lothrop, who diagnosed the case and made exploration. A few days later, when it came under his care, this opening had healed; a large, free, crucial incision was made, and about eight ounces of thick gumous blood discharged. In about three weeks the case resulted in complete recovery.

*Dr. Miner* called the attention of the Association to the treatment of hip joint disease and deformities by extension and counter extension, and exhibited an elastic extension splint received from Dr. H. G. Davis of New

York City. He had had, some experience in this manner of treatment and should be only speaking the unanimous sentiment of the profession when he said that it was an efficient means of removing deformity from contracted muscles, and at the same time was often useful in relieving pain and arresting the progress of diseased action. He believed from examination of testimony upon the subject that Dr. H. G. Davis was entitled to the honor of introducing to the profession this manner of treating deformities and diseased joints by what he calls "elastic extension." Drs. Sayre, Andrews, Hamilton, Taylor, E. S. Cooper, Olcott, Vedder and others, had invented modifications of Dr. Davis' splint, while the principles of treatment remained the same. He had seen the statement in a review of Dr. Richard Barwell's Treatise on Diseases of the Joints, in the *American Medical Monthly for December*, 1861, that the author of that very valuable book had failed to acknowledge the claims of Dr. Davis, which are now conceded very generally by American Snrgeons. The reviewer says: "We are pleased to find Dr. Barwell follows the right path; we are disappointed to find that he is not fully conscious of the principles upon which successful treatment depends; that his appliances are inferior, and that he does not even mention the name of their originator."

The original design of the instrument and the first object of the plan was to relieve or arrest diseased action in morbus coxarius. It has since been extended to the treatment of Potts' disease of the spine, scrofulous disease of the knee, and diseases of the joints generally. It had been claimed by some of the surgeons who had modified Davis' splint that they were entitled to the credit of discovery, and a committee had been appointed by the Section on Surgery of the New York Academy of Medicine, to investigate and make report upon the rival claims. This committee had been unanimous in awarding to Dr. Davis this honor. It consisted of Prof Alfred Post, Dr. Gurdon Buck and Prof. Willard Parker.

*Dr. White* remarked, that the great object proposed was to make extension, and this was accomplished by the improved splints now in use. The patient could wear it, and yet was not confined; could walk and ride with considerable comfort, while yet the extension was kept constantly applied. Had recently advised in consultation in case of hip disease, Dr. Sayre's splint, which was applied, and answered the indications remarkably well. The greatest difficulty was to prevent excoriation in the groin. This was represented to Dr. Sayer, who replied that he now used a large rubber tube for making elastic counter-extension. This suggestion was adopted with but partial success; yet the result of the treatment was most satisfactory. The old fashion treatment of applying extension, by means of a weight over a pully was equally good, with the exception that it necessitated confinement. Did not desig. to say who was the first inventor of the plan; so far as he knew, it was Prof. Moore, who, he did not think had ever made any claims to originating it. Thought that a surgeon of Dr. Sayer's standing and attainment would claim nothing which did not rightfully belong to him. Had the pleasure of a personal acquaintance, and said the profession was indebted to Dr. Sayer for valuable suggestions in the treatment of diseases of the joints and deformities. The object of the treatment in disease of the joint, was to remove pressure, relieve any deformity, and at the same time allow the patient healthful exercise. By continued extension the contraction of muscles and consequent deformity was overcome, pressure was relieved by separating the heads of the bones, and patients could even walk with comparative comfort. By thus relieving the pressure from contraction of muscle and weight of body, diseased surfaces would take on healthy action and pain was often greatly lessened.

*Dr. Miner* desired to express no personal opinions concerning the claims of Drs. Davis and Sayer, and had simply quoted the report of the committee, or remarks of the chairman, who said, in speaking upon this point, that "but for Dr. Davis the profession would have known nothing about it." That such an opinion was correct, or the testimony which was presented to them truthful and satisfactory, he had no means of knowing, but was willing to presume in the absence of any conflicting evidence, that Dr. Post was justified when he tells the Academy of Medicine "that the honor of this discovery belongs wholly to Dr. Davis." In saying this he did not desire in the least to disparage any rightful claims of Dr. Sayer, or others who may have claimed improvements. There were two other points suggested by the remarks of Prof. White which he desired briefly to notice, though he feared some difficulty in making himself clearly understood.

In morbus coxarius the thigh is generally flexed upon the pelvis, and the leg upon the thigh, the knee projecting prominently forward; at least it is so most commonly in the second and third stages of the disease. When in the third stage of the disease if the acetabulum has suffered severely the toes incline inwards, as in dislocation of the thigh-bone upwards upon the dorsal surface of the ilium, and as before remarked the leg is flexed upon the thigh. This is produced by the rigid contraction of the flexor muscles of the thigh and leg. If attempts at extension are made by extending rod, or other means, the femur becomes the lever, the acetabulum the fulcrum, and the contracted muscles the resisting force; while if the knee is the diseased joint and we extend the leg while the ham-string tendons offer resistance, the tibia is the lever, the articulating surface of the femur the fulcrum, and the contracted muscles the resisting force. These propositions appeared to him so plain and conclusive as not likely to be contested. It had been virtually said by Dr. White, that extension, acting upon these bones relieved the pressure, and thus also afforded relief of pain and often arrested diseased action; whereas he proposed to show on the contrary that the pressure is not only not relieved, but is greatly increased, until such relaxation of the muscles is obtained as to allow the bones a straight position, until the deformity is removed, the muscular contraction overcome; and that, when this is obtained, the articulating surfaces of the bones are not separated by any force of extension which it is practicable to adopt. Whoever has attempted the restoration of the leg after contraction of the flexor muscles, by either gradual or forcible extension, could not have failed to observe the immense pressure brought to bear upon the articulating ends of the femur and tibia. The resistance of the muscles is always very great, and in most cases so great as not to yield, but force the head of the tibia backwards, producing partial dislocation. This condition of the joint and the forces by which it is produced, has been described by John Erichsen, Esq., of England, and published in the London Lancet for July, 1856, and an instrument has been invented by his mechanic, by which this increase of pressure might be avoided by applying an extending force above as well as below the insertion of the ham-string muscles, and thus, as he claims, restore the line of the leg, without producing the backward displacement. He has illustrated the true line of forces active in the restoration of such deformities, but has not told us, in so many words, that without his new power drawing upon the upper end of the bone the pressure was increased by extension, as applied to any one point below the resisting force, but he probably presumed we should know it without being told.

In making extension upon the femur while it is flexed upon the pelvis, the same principle is plainly in action, and the head of the thigh-bone is pressed harder by the extending force until the deformity is removed, that is, until the contracted muscles have so far relaxed as to destroy the action of the lever. Upon this principle is based the mode of reducing dislocation of the head of the femur by manipulation, and when no reasonable force of extension will restore it in place, the thigh is *flexed* strongly upon the pelvis, and the head of the thigh-bone *lifted* over the edge of the acetabulum. We have very much the same condition of muscular contraction in morbus coxarius, which is often produced in dislocation of the head of the thigh-bone, and extension in either case produces increased pressure upon the head of the bone and the acetabulum or ilium. The almost positive impossibility of reducing this dislocation by extension, in many instances is satisfactorily explained only upon the ground that the muscles offer resistance to all the extending force it is practicable to employ, and thus force the dislocated head closely down upon the ilium, and will not allow its raising over the edge of the articulating cup. When this force of contracting muscle is yielded to by still more complete flexion, the pressure is relieved and the object is readily accomplished. *Flexion* of the thigh then (when the flexor muscles are irritated and contracted,) and not extension, is the only rational method of relieving the pressure.

While the pressure is at first increased upon the articulating surfaces of contracted joints by extension, it is changed to a healthy place of bearing or to a less actively diseased one, and this, together with the perfect rest which is obtained would usually explain the relief from pain which sometimes follows extension; and much of the benefit which we derive from application of extending splints, was due to placing the limb in more natural position, overcoming muscular contraction and changing the pressure from a diseased to more healthy surface. Upon the point of separating the heads of bones after muscular contraction has been overcome, he would only say, that the anatomical structure of joints, the great power of muscular action in resisting any unnatural traction, and the great difficulty in making any strong continued extension, without painful pressure and unnatural force, constitute sufficient grounds for doubting the position, that bones are separated by extension.

*Dr. White* would like to know how a change of bearing could take place in the cup-like cavity of the acetabulum, which in hip-joint disease is supposed to be affected sooner or later in its whole surface? Did not think the thigh was generally flexed upon the pelvis to the extent represented by Dr. Miner, and could not see how gradual extension could produce increase of pressure, but thought that it would overcome muscular contraction and greatly relieve the diseased surfaces from pressure.

Would also inquire how it should be so impossible to separate the bones when at one stage of the disease the leg is elongated, sometimes one or two inches? Thought if the diseased action could lengthen the leg, gradual extension might do it. Had no doubt gradual extension would lessen the pressure at the articulation.

*Dr. Miner* thought it sufficiently plain, how *any* force brought to bear in a new direction upon the muscles of a diseased hip would change the place of pressure, while if all the structures of the articulation were equally involved in diseased action, such cases would prove exception so far as pressure upon less diseased surfaces formed the ground of relief from pain. It would probably also form an exception to the rule of obtaining freedom from pain by extension.

If at any stage of morbus coxarius the leg was actually lengthened, it was only in slight degree by deposit of inflammatory product, crowding the head of the femur from the cup-like cavity of the articulation. It was however probable that there was rarely if ever any actual lengthening of the leg, and only an apparent elongation from change in the position of the pelvis; but admitting this, it would constitute no evidence that any artificial force could produce similar result.

*Dr. White* could only reply in the language of the ancient woman when informed that St. Paul did not teach such doctrine, who replied, "St. Paul and I disagree upon that subiect." The literature of the profession was opposed to Dr. Miner's hypothesis.

*Dr. Miner* replied that if the literature of the profession was in opposition, which he did not acknowledge, he had rather be right, and alone, than wrong, supported by Paul and all the Apostles, and was all the more anxious to show the philosophy and soundness of the ground he had taken.

*Dr. Wyckoff* related the case of a young lad of well marked scrofulous habit who, two years since, had taken cold; the thigh became flexed upon the pelvis, and three months after an abscess opened and had discharged ever since. Upon the ilium was a place where the bone is evidently absent, having been carious. For a long time could not walk except with crutch; afterwards the contraction abated, and he had been able to attend to business for the last year, walking as usual without trouble upon the leg.—About one week since he commenced to have pain in the hip, which increased until he was again laid up; no other evidence of hip joint disease. The decayed place on the ilium is plainly to be observed. His object in mentioning the case is to inquire if extension would be beneficial under uch circumstances.

*Dr. Miner* replied, that upon the supposition of possible incipient disease of the joint, which as yet gave no positive symptoms, extension might be useful, preventing deformity, insuring rest, and relieving pain.

*Dr. White* could see no indications for applying extension in the case related by Dr. Wyckoff. And since he had once previously recovered the full use of the leg and joint, after similar attack, showing freedom from disease of joint at that time, should regard the application of extension as unnecessary.

*Dr. Gay* remarked that he had been greatly interested in the ingenious theory proposed by Dr. Miner. If he is correct, the practice of Dr. Davis based upon the idea of relieving pressure by extension necessarily falls; could not conceive of a diseased joint being benefited by increase of pressure. If this theory is correct he could invent a better instrument for diseased joints than Dr. Davis'. Seemed to him that *separating* the bones was the only way of properly treating such cases.

The Association adjourned to Tuesday evening, January 7, 1863.

J. F. Miner, Secretary.

